# Draft genome sequence of *Trichosporon asahii OK01* isolated from a cystic fibrosis patient in Oklahoma

**DOI:** 10.1128/mra.00291-26

**Published:** 2026-07-21

**Authors:** Noopur Dasgupta, Rebecca Wilson, Chelsea L. Murphy, Kaylea M. Feldman, William C. Starr, Elizabeth Pascual, Erika I. Lutter

**Affiliations:** 1 Department of Microbiology and Molecular Genetics, Oklahoma State University 7618, Stillwater, Oklahoma, USA; 2 High-Performance Computing Center, Oklahoma State University 7618, Stillwater, Oklahoma, USA; Rochester Institute of Technology, Rochester, New York, USA

**Keywords:** fungi, *Trichosporon*

## Abstract

*Trichosporon* species cause potentially life-threatening localized and disseminated infections in immunocompromised individuals. While fungal lung infections in cystic fibrosis patients are well established, the significance of *Trichosporon* species is not widely recognized. Herein, we describe the genome of *Trichosporon asahii* isolated from a cystic fibrosis patient in Oklahoma, USA.

## ANNOUNCEMENT

Cystic fibrosis (CF) is a genetic disease that leads to persistent infection by a variety of pathogens, including bacteria and fungi. Over the last few years, novel potential pathogens have been recovered from CF patients’ sputa with greater frequency, including *Trichosporon* species (spp.) (1). Many case studies over the last decade have identified *Trichosporon* spp. in CF respiratory sections (2) and suggest that infection may contribute to the deterioration seen in CF patients (3, 4).

CF sputa samples were collected from the Pulmonary and Cystic Fibrosis Clinic at Oklahoma Children’s Hospital in Oklahoma City, OK, USA (institutional review board exempt). Samples were plated on selective media, including nutrient agar (NA), incubated at 37°C for 96 h, and all microbial growth was stored as a mixed culture in 10% skim milk at −80°C. Screening for multidrug-resistant microbes identified isolate *Trichosporon asahii* OK01 resistant to ciprofloxacin, oxacillin, and polymyxin B from a mixed culture grown originally on NA. This isolate was originated from the sputa sample obtained from a 38-year-old male patient (no exacerbation at the time of sputa collection) on 6 March 2014.

Genomic DNA was isolated from *T. asahii* OK01 using the ZR Fungal/Bacterial DNA Miniprep Kit (Zymo Research Corporation, Irvine, CA, USA). DNA concentration and purity were checked on a NanoDrop OneC Microvolume UV-Vis Spectrophotometer, verified by agarose gel electrophoresis, and submitted to SeqCenter (Pittsburgh, PA, USA) for genome sequencing using their Illumina Whole Genome Sequencing protocol. DNA extractions at SeqCenter used the Zymo BIOMICS DNA Miniprep Kit. Raw sequencing reads were assembled using the tagmentation and PCR-based Illumina DNA Prep Kit. Sequencing was performed on an Illumina NovaSeq 6000 platform, generating 2 × 151 bp paired-end reads. Quality control was carried out using bcl-convert, and a total of 8,268,848 reads were obtained, corresponding to an average sequencing depth of 51.4×. (5). Short read assembly was performed with Unicycler (v 0.4.8) (6), and assembly statistics were computed with QUAST (v 5.0.2) (7). The assembly was identified as *Trichosporon asahii* via a BLAST homology search of all contigs against the National Center for Biotechnology Information (NCBI) nt database (July 2023) (Table 1). The completeness of the genome was assessed using BUSCO version 5.8.2, using the Tremellomycetes odb12 database.

**TABLE 1:** Detailed statistics of the sequencing and assembly

Parameters	Values
Total Size (Mbp)	24.31
Coverage	42.5x
# contigs	113
N50	414,355
L50	18
%GC	59.59
% Completeness	95.10
SUBID	SUB16038622
NCBI BioProject	PRJNA1431409
NCBI BioSample	SAMN56309270
NCBI accession	JBVVWQ000000000
Organism	*Trichosporon asahii* OK01

**Fig 1:**
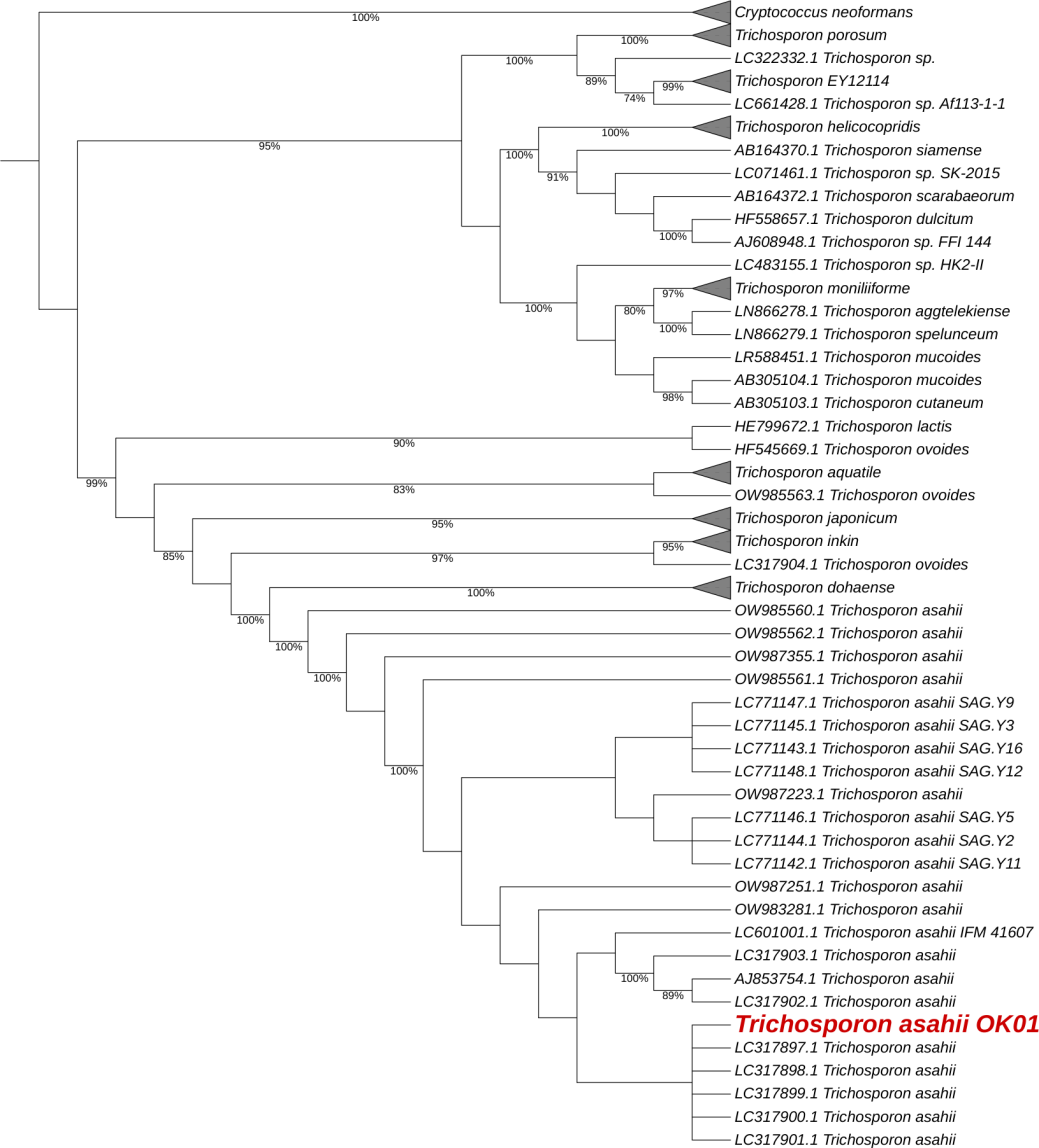
Phylogenetic tree.

To verify the taxonomy, *Trichosporon* sequences containing the genes for 18S ribosomal RNA (rRNA), ITS1, 5.8S rRNA, ITS, and 28S rRNA were downloaded from NCBI (September 2023), and the corresponding genes were extracted via BLASTn. Sequences were aligned with Clustal-Omega (v 1.2.3) (8), and an approximately maximum-likelihood phylogenetic tree was constructed with FastTree (v 2.1.10 SSE3) (9) (Fig. 1). The tree was rooted with *Cryptococcus neoformans* sequences and visualized in iTOL (10). Default parameters were used for all programs. The final assembly comprises 113 contigs for a total length of 24,304,609 bp. The L50 of the assembly is 18, and the N50 is 414,355 bp.

## Data Availability

This Whole Genome Shotgun project has been deposited at DDBJ/ENA/GenBank under the accession number JBVVWQ000000000. The version described in this paper is JBVVWQ010000000. The raw sequence reads are available in the Sequence Read Archive under the BioProject accession number PRJNA1431409.
